# Supplementary material for: A Potential Predictive Role of the Scalp Microbiome Profiling in Patients with Alopecia Areata: Staphylococcus caprae, Corynebacterium, and Cutibacterium Species
Source: Microorganisms. 2022 Apr 21;10(5):864. doi: 10.3390/microorganisms10050864 (PMC9148078; doi:10.3390/microorganisms10050864)
Supplement: Supplementary file 1 [file microorganisms-10-00864-s001.zip › microorganisms-1672440-supplementary.pdf]

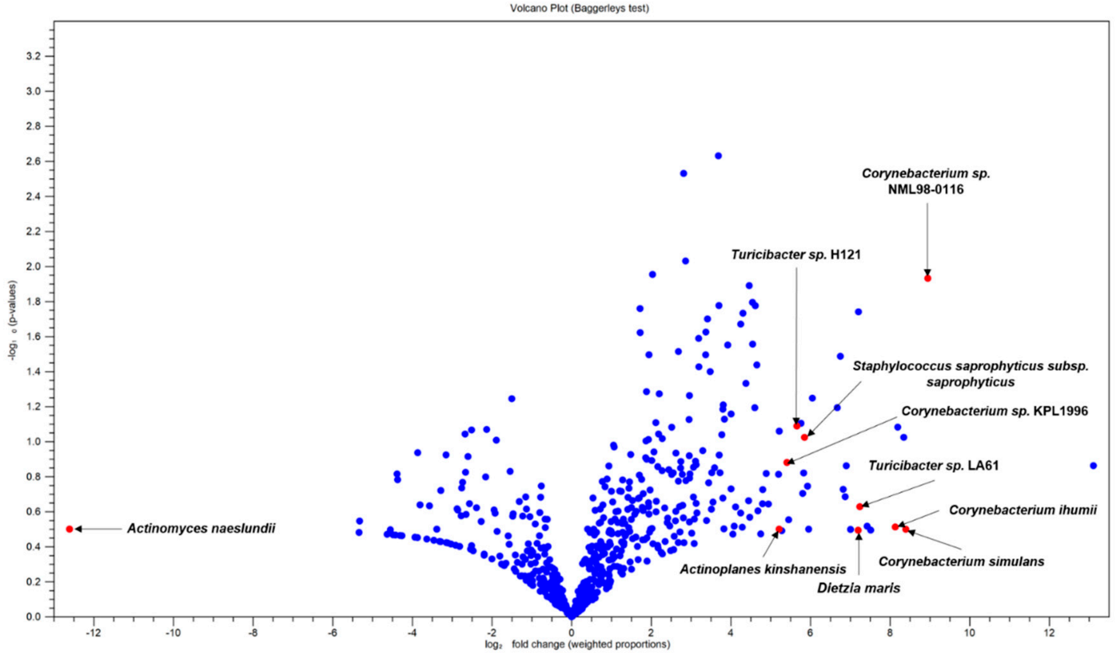

Supplementary Figure 1. Volcano plot showing bacterial taxa related to alopecia areata. The genus *Corynebacterium* which is represented in the microbiome by the several OTU: *Corynebacterium ihumii, Corynebacterium simulans, Corynebacterium* sp. NML98-0116, *Corynebacterium* sp. KPL 1996 were distinctive to AA than HC.
